# Does Caesarean Section or Preterm Delivery Influence TGF-β2 Concentrations in Human Colostrum?

**DOI:** 10.3390/nu12041095

**Published:** 2020-04-15

**Authors:** Bożena Kociszewska-Najman, Elopy Sibanda, Dorota M. Radomska-Leśniewska, Karol Taradaj, Patrycja Kociołek, Tomasz Ginda, Monika Gruszfeld, Ewa Jankowska-Steifer, Bronisława Pietrzak, Mirosław Wielgoś, Jacek Malejczyk

**Affiliations:** 1 Department of Neonatology, Medical University of Warsaw, 02-091 Warsaw, Poland; monikagruszfeld@o2.pl; 2 Asthma, Allergy and Immune Dysfunction Clinic, Twin Palms Medical Centre, 113 Kwame Nkrumah Avenue, Harare, Zimbabwe; ensibanda@gmail.com; 3 Department of Pathology, Medical School, National University of Science and Technology, PO Box AC 939, Ascot, Bulawayo, Zimbabwe; 4 Division of Pathophysiology and Allergy Research, Medical University of Vienna, 1090 Vienna, Austria; 5 Department of Histology and Embryology, Centre of Biostructure Research, Medical University of Warsaw, 02-004 Warsaw, Poland; dradomska@wum.edu.pl (D.M.R.-L.); ewa.jankowska-steifer@wum.edu.pl (E.J.-S.); jacek.malejczyk@wum.edu.pl (J.M.); 6 Students Scientific Research Group by Department of Neonatology, Medical University of Warsaw, 02-091 Warsaw, Poland; karol.taradaj@wum.edu.pl (K.T.); patrycja.kociolek96@gmail.com (P.K.); ginda.tomek@gmail.com (T.G.); 7 Department of Obstetrics and Gynaecology, Medical University of Warsaw, 02-015 Warsaw, Poland; bpietrzak@wum.edu.pl (B.P.); miroslaw.wielgos@wum.edu.pl (M.W.)

**Keywords:** human colostrum, human milk, immune modulators, TGF-β, cytokines, health outcomes, chronic disease, immunological outcome

## Abstract

Human colostrum (HC) is a rich source of immune mediators that play a role in immune defences of a newly born infant. The mediators include transforming growth factor β (TGF-β) which exists in three isoforms that regulate cellular homeostasis and inflammation, can induce or suppress immune responses, limit T helper 1 cells (Th1) reactions and stimulate secretory immunoglobulin A (IgA) production. Human milk TGF-β also decreases apoptosis of intestinal cells and suppresses macrophage cytokine expression. The aim of the study was to determine the concentration of TGF-β2 in HC obtained from the mothers who delivered vaginally (VD) or by caesarean section (CS), and to compare the concentrations in HC from mothers who delivered at term (TB) or preterm (PB). In this study, 56% of preterm pregnancies were delivered via CS. The concentrations of TGF-β2 were measured in HC from 299 women who delivered in the 1st Department of Obstetrics and Gynaecology, Medical University of Warsaw: 192 (VD), 107 (CS), 251 (TB), and 48 (PB). The colostrum samples were collected within 5 days post-partum. TGF-β2 levels in HC were measured by the enzyme-linked immunosorbent assay (ELISA) test with the Quantikine ELISA Kit-Human TGF-β2 (cat.no. SB250). Statistical significance between groups was calculated by the Student *t*-test using StatSoft Statistica 13 software. The mean TGF-β2 concentration in patients who delivered at term or preterm were comparable. The levels of TGF-β2 in HC were higher after preterm than term being 4648 vs. 3899 ng/mL (*p* = 0.1244). The delivery via CS was associated with higher HC concentrations of TGF-β2. The levels of TGF-β2 were significantly higher in HC after CS than VD (7429 vs. 5240 ng/mL; *p* = 0.0017). The data from this study suggest: caesarean section was associated with increased levels of TGF-β2 in HC. The increased levels of TGF-β2 in HC of women who delivered prematurely require further research. Early and exclusive breast-feeding by mothers after caesarean section and premature births with colostrum containing high TGF-β2 levels may prevent the negative impact of pathogens which often colonize the gastrointestinal tract and may reduce the risk of chronic diseases in this group of patients.

## 1. Introduction

The correlation between breastfeeding and the health of the newborn as well as predispositions to infectious diseases, allergic or autoimmune health is an issue which has been mentioned in many cultures for thousands of years [1,2]. There is a lot of research suggesting not only decreased risk of gastrointestinal, communicable and non-communicable diseases but also reduced prevalence of chronic diseases in the subsequent life of newborns who were breastfed [3,4,5,6,7,8]. It was also reported that breastfeeding can reduce asthma prevalence in children, but its correlation with allergic diseases still remains unclear [9]. Those studies confirm the immunomodulatory and protective function of human milk and the importance of breastfeeding. The composition of human milk (HM) changes with the phase of lactation. It may also be different depending on maternal origin [10]. We differentiate three types of HM: colostrum, transitional milk, and mature milk.

Human colostrum (HC) is the form of milk produced within the first days postpartum. It is a very rich source of nutrients for growth and development of a newborn, including immune mediators such as hepatocyte growth factor (HGF), epidermal growth factor (EGF), insulin-like growth factor I and II, immunoglobulins, transforming growth factor β (TGF-β) and a lot more [7,11]. There are three isoforms of TGF-β in HC: TGF-β 1, 2 and 3, with TGF-β2 being the most abundant, accounting for up to 95%. The concentration of TGF-β changes during lactation with the highest level in the colostrum [12,13,14]. TGF-β regulates IgA production and induces oral tolerance. There are animal studies suggesting that TGF-β is the most important in the neonatal period, when the endogenous production of the cytokine is still inadequate and when new antigens are being introduced to the gastrointestinal tract. According to this, authors suggest that TGF-β in maternal milk may prevent early sensitization and atopic diseases during breastfeeding [13]. TGF-β also regulates lymphocytes’ T responses, leading to better gut tolerance [7]. Furthermore, TGF-β2 can inhibit the inflammatory response in immature human intestinal epithelial cells and suppress the expression of macrophage cytokines, thereby reducing the risk of necrotizing enterocolitis (NEC) in preterm neonates [15,16,17]. On the other hand, there are also data suggesting that higher levels of TGF-β2 may be related to NEC [18]. Furthermore, systemic review from 2019 suggests an increased risk of allergic diseases in infants whose mothers had higher levels of TGF-β2 in milk, but due to differences in studies’ methodology and outcomes, this issue remains unclear [19]. Recent studies showed also the correlation between the concentration of TGF-β2 in HC and diversity of gut microbiota [20]. It has been already established that activation of tissue macrophages due to the surgery induces TGF-β1 synthesis [21]. There are also known biological effects of TGF-β family proteins that include control of cell proliferation, differentiation, and control of wound healing [22]. Those findings may explain the changes of TGF-β2 concentration in the colostrum obtained from mothers who delivered via caesarean section. However, this issue remains unclear. The aim of our study was to determine the concentration of TGF-β2 in HC obtained from mothers who delivered vaginally (VD) or by caesarean section (CS), and to compare the concentration of TGF-β2 in HC in term (TB) and preterm (PB) births.

## 2. Materials and Methods

### 2.1. Design

A prospective study of patients who delivered at 1st Department of Obstetrics and Gynaecology, Medical University of Warsaw, Poland.

### 2.2. Study Population

Two hundred ninety-nine mothers were included in the study. The women were between 19 and 44 years old. The newborns were born between the 25th and 41st week of gestation (WG). Mean weeks of gestation in specific patient subgroups—term delivery, preterm delivery, caesarean section and vaginal delivery—were respectively 26 ± 5; 26 ± 4; 27 ± 5; 25 ± 4.

The patients received uniform analgesic pharmacotherapy with paracetamol according to obstetric guidelines. The type of anaesthesia through caesarean section as well as for vaginal delivery was performed in accordance with the single mandatory midwife standard, whether it was a premature birth or at the time. Anaesthesia for caesarean section: all patients were given subarachnoid anaesthesia (spinal anesthesia). The administered drugs included: 1.5–3 mL 0.5% bupivacaine and 20–25 µg of fentanyl. On a day zero, after surgery, the patients received 10 mg of morphine every 4–6 h subcutaneously. On the first and sometimes on the second day, the patients were given paracetamol at a maximum dose of 1 g every 6 h. Anaesthesia for vaginal delivery: all patients the following were given epidural anaesthesia (epidural anesthesia). The drugs used included: 2–3 mL 0.5% bupivacaine with adrenaline and 100 µg of fentanyl. On the first and sometimes on the second day, the patients were given paracetamol at a maximum dose of 1 g every 6 h.

Both after vaginal delivery and after caesarean section, the patients did not take any drugs on the day of colostrum collection (3 days after the birth). The generally available pharmacokinetic properties of the used drugs, including their biological half-life and excretion mechanisms, prove that neither the drugs nor their active metabolites were found in the blood or colostrum on the day of the colostrum collection.

The inclusion criteria for the study were sufficient lactation for collect samples in mothers and written informed consent for participation in the study which was obtained at the postnatal ward. The criterion of exclusion was the presence of an active inflammatory process, inability to express colostrum or lack of mother’s consent. The inclusion and exclusion criteria are presented in Table 1. During the investigations, they were divided into the following groups: mothers of preterm (<37 WG) and term (≥37 WG) neonates, mothers who delivered vaginally (VD) or via elective caesarean section (CS). The data describing mothers and newborns were taken from medical records.

### 2.3. Collection of Human Colostrum (HC)

Human colostrum samples (15–20 mL) were collected into single-use, sterile tubes made of polypropylene by either manual expression or the use of a breast pump between the 3rd and 5th post-partum days. Colostrum was collected on the 3rd day after delivery from patients who delivered at term (251 samples) and patients who born late preterm newborns (39 samples). In the group of patients who delivered extremely preterm newborns, 2 samples were taken on the 4th day after delivery and 1 on the 5th day after delivery. In the group of patients who delivered very preterm newborns, samples were taken: 1 on 3rd day, 4 on 4th day and 1 on 5th day after delivery. The samples were centrifuged at 500× *g* for 10 min at 4 °C. The lipid layer and the cell pellet were discarded, and the clear colostrum samples were harvested and aliquots were prepared. The cell-free aqueous fractions were stored at −80 °C in portions to avoid freeze-thaw cycles and were used for TGF-β2 evaluation.

### 2.4. Quantification of Transforming Growth Factor β (TGF-β2) in Human Colostrum

The concentrations of TGF-β2 in human colostrum were evaluated using a commercial kit Quantikine Human TGF-β2 Immunoassay (R&D Systems, cat. No. SB250, Minneapolis, MN, USA) according to the manufacturer’s instructions. The Immunoassay is a 4.5 h solid phase enzyme-linked immunosorbent assay (ELISA) designed to measure active TGF-β2 in the cell culture supernatant. It contains recombinant human TGF-β2 expressed by Chinese hamster ovary (CHO) cells and has been shown to quantitate the recombinant factor accurately. To activate the latent TGF-β2 form, each sample was acidified by 1N HCL for 10 min which was followed by neutralization by 1.2N NaOH/0,5M 4-(2-Hydroxyethyl)piperazine-1-ethanesulfonic acid (HEPES). This method activates all of the TGF-β2 in a sample. Since samples have been diluted in the activation step, the concentration read from the standard curve was multiplied by the dilution factor, 7.8. All standards and samples were tested in duplicate and the mean values were used for calculation.

### 2.5. Statistical Analysis

The data analyzed using the Student *t*-test were represented as the mean ± standard deviation (SD), while those obtained from the Mann-Whitney-U analyses were presented as the medians ± IQR (interquartile ranges). The differences between the groups were evaluated by the Student *t*-test and the Mann–Whitney U test using Statistica StatSoft 13 software (Tibco Software Inc., Palo Alto, CA, USA). The differences were considered significant at *p*-value < 0.05.

### 2.6. Ethical Approval

The study was approved by the Bioethical Committee of the Medical University of Warsaw and all patients signed an informed consent to participate in the study.

## 3. Results

### 3.1. Description of Study Participants

A total of 299 women fulfilled the inclusion criteria and were enrolled. Their mean age was 26 years and their ages ranged between 19–44 years. They were all healthy non-smokers with no known chronic diseases. Among them, 192 (64.2%) mothers had delivered vaginally (VD), 107 (35.8%) delivered by caesarean section, (CS). The gestational ages at the time of delivery were at term (TB ≥ 37 weeks) for 251 (84%) mothers while 48 (16%) delivered preterm infants (PB; <37 weeks). Fifty-six percent (56%) preterm infants were delivered by CS.

### 3.2. Measurements of TGF-β2 Concentrations

The concentration of TGF-β2 was measured in HC from all the 299 women. The results obtained fulfilled the testing requirements of the kit manufacturer. The concentrations of natural TGF-β2 from colostrum supernatants showed linear curves that were parallel to the standard curves obtained using the recombinant kit standards. The analysis of the statistical significance of the differences between the concentration of the TGF–β2 in the colostrum based on the gestational age and modes of delivery was conducted.

### 3.3. Gestational Age at the Time of Delivery

**Figure 1:**
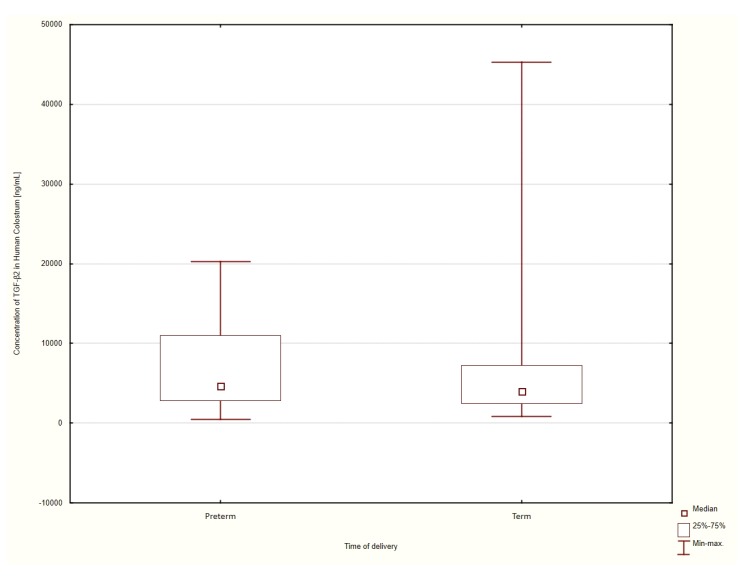
Comparison of the median TGF-β2 concentration in the human colostrum of patients had delivered at term and preterm.

The patients were considered to have delivered at term if the delivery occurred at a date ≥37 weeks of gestation, patients who delivered at gestational ages <37 weeks were classified as pre-term. The concentrations of TGF-β2 in the colostrum were measured for all the enrolled women. When categorized by gestational age at the time of delivery, the median concentration of the TGF-β2 cytokine who had delivered at term was 3899 ± 533 ng/mL. The concentrations of colostrum TGF-β2 amongst those who delivered preterm were 4648 ± 772 ng/mL. The data were analyzed using the Mann–Whitney U test for non-parametric variables. Although the concentration of TGF-β2 in the colostrum of patients who delivered preterm was higher, the differences were not statistically significant (*p* = 0.1244). The results are shown in Figure 1.

The difference in mean TGF-β2 levels after preterm delivery was analyzed depending on the date of delivery. The results are shown in Table 2. Due to the small number of samples collected from patients who delivered extremely preterm: ≤28 WG (*n* = 3), very preterm: 29–32 WG (*n* = 6), and the significant prevalence of samples of patients who delivered late preterm: 33–36^6/7^ WG (*n* = 39), it is not possible to present statistically significant differences between these groups.

### 3.4. Mode of Delivery

The mode of delivery of the infants was vaginal delivery or caesarean section. The concentrations of the colostrum TGF-β2 was compared between the two groups.

**Figure 2:**
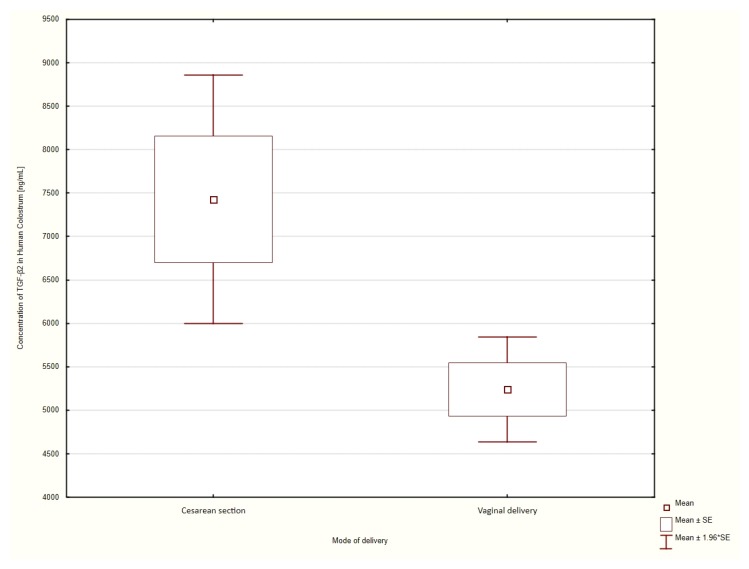
Comparison of mean TGF-β2 values in patients who had delivered via caesarean section or who delivered vaginally.

The average concentration of the TGF-β2 cytokine in the colostrum of patients who delivered vaginally was 5240 ± 308 ng/mL compared to 7429 ± 730 ng/mL in patients who delivered surgically. Statistical analyses using the Student *t*-test showed that the concentration TGF-β2 in the colostrum of patients who had delivered by CS was significantly higher than in those who delivered vaginally (*p* = 0.0017). (Figure 2).

#### Term Delivery

**Figure 3:**
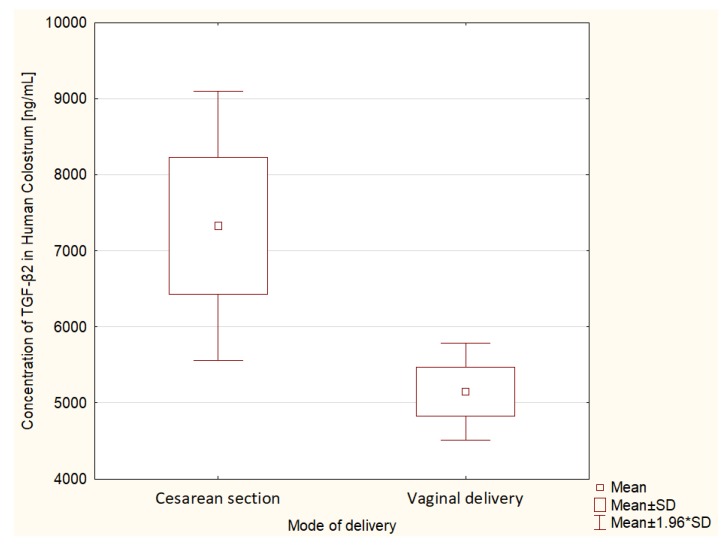
Comparison of mean TGF-β2 values in patients who had delivered at term depending on the mode of delivery.

An additional analysis of differences in TGF-β2 concentration in HC was performed specifically in a group of 251 patients who delivered at term. The average concentration of TGF-β2 in HC in patients who delivered surgically was 7329 ± 8018 ng/mL. The mean TGF-β2 in HC in patients who delivered vaginally was lower and amounted to 5148 ± 4039 ng/mL. There was a statistically significant difference in the t-student test in the concentration of TGF-β2 depending on the type of delivery in the group of patients who delivered at term (*p* = 0.006). The results are shown in Figure 3.

## 4. Discussion

The term colostrum describes the first secretions of mammalian mammary gland following birth. Colostrum is highly nutritious and is fortified with abundant proteins that play a pivotal role in non-specific immune responses. HC contains many types of immunological agents including secretory IgA antibodies, antimicrobial agents such as lactoferrin, lysozyme, and lactoperoxidase anti-inflammatory factors, immunoregulators, living activated leukocytes and others [23]. These defense agents protect the mucosa of the alimentary and respiratory tracts from microbial pathogens. One of the growth factors that promotes the secretion of IgA and is TGF-beta. TGF-beta is a crucial component of the innate defence mechanisms. It inhibits intestinal inflammation, enhances oral tolerance and reduces the risk of potentially fatal necrotizing enterocolitis. Human colostrum contains an immune system that compensates for the developmental delays in the infant’s immune system. The cytokine has the propensity to promote an immunological pathway that promotes the development of atopic dermatitis [24] and autoimmunity in later life [25]. TGF-β is highly regulated due to its important function in the body. The mechanism of TGF-β ligands bioavailability regulation involves releasing of the growth factor from cells and modulation, inhibition, activation or enhancing the binding TGF to its receptors [26]. TGF-β is secreted from cells as a large latent complex (LLC) formed by three components: TGF-β, LAP (latency-associated protein), and LTBP (latent TGF-β binding proteins). LTBT assists in the conversion of the latent TGF-β to its active form [26,27]. The latency components function as natural antagonists of TGF-β activity, to target TGF-β to distinct tissues, and to maintain a reservoir of TGF-β. The activation process allows binding to its high-affinity receptor. It is a critical step in the control of TGF-β activity [27].

In our study, we have used the most common activation method of TGF-β conversion from latent to its active form i.e., acidification and neutralization of the studied material. This method lowers the pH to 2.0 for a short period of time and activates all of the TGF-β in a sample and allows the active/mature form of TGF-β to be measured [27].

In this study, human colostrum was obtained from 299 otherwise healthy women and the concentrations of the dominant TGF-β2 fraction were analyzed using standard ELISA techniques, performed by laboratory personnel. The variables that were analyzed were the association of cytokine concentrations in colostrum and the gestational age at delivery and mode of delivery. Our results show that the concentrations of TGF-β2 differed in the case of each of those variables. The abundance of TGF-β2 in human colostrum has been previously reported and shown to be significantly associated with the induction of serum IgA production [28] that plays a pivotal role in innate defenses at all mucosal surfaces including the gastrointestinal, respiratory and urogenital tracts. The concentration of the TGF-β2 isoform is higher in the colostrum and milk collected in the first week after delivery from mothers of food-tolerant neonates compared to the milk of mothers of food intolerant neonates [18]. This finding supports the role of TGF-β2 in intestinal homeostasis. Although the mechanisms are unclear, these appear to be the examples of TGF-β2 deploying secretory immunoglobulin A (sIgA) to protect mucosal surfaces. The implication is that the protective proteins are deployed at higher concentrations depending on the stage of maturation of the mucosal immune response mechanisms wherein sIgA plays a critical role.

The concentration of TGF-β2 was higher in preterm colostrum than in term colostrum, although the median concentrations were not statistically significant. In our study, we did not consider the total amount of colostrum from women who delivered prematurely produced per day. In this case the daily volume of production is much smaller than in women who deliver after a normal length of pregnancy. When that is done, it would be possible that the total amount of TGF-β2 in colostrum produced per day may not be significantly different. However, there is also a study in which significantly higher amounts of active and latent TGF β2 in preterm than full-term milk were detected. The authors showed that preterm human milk showed minimal TGF-β bioactivity in the native state but contained a large pool of latent TGF-β. TGF-β2 was the predominant isoform of TGF-β in preterm milk. The authors showed that the addition of bacterial neuraminidase to preterm human milk increased TGF-β bioactivity. Preterm milk contains large quantities of TGF- β, but most of it is in an inactive state. Addition of neuraminidase can increase TGF-β bioactivity in preterm milk and enhance its anti-inflammatory effects [29]. This isoform is intrinsically produced throughout the body and is involved in cell proliferation, cell differentiation, cell motility, programmed cell death and the suppression of tumorigenesis. Higher levels in preterm colostrum than in term milk could suggest compensation for the immaturity of these functions in the neonate. Enterally administrated TGF β2 may, for example, protect preterm neonates against NEC, but this requires further investigation.

In our study, TGF-β2 concentrations were high in preterm as well as in children born via caesarean section. Therefore, it is possible that elevated levels of TGF-β2 in mothers during the first post-partum week resulted from surgery rather than gestational ages. The study did not investigate the contributions of neonate prematurity or maternal surgical wound healing, both of which are associated with increased levels of the TGF-β1 isoform. The differentiation of the triggers of TGF-β2 production in this scenario is complex because of the fact that 56% of preterm deliveries were via the surgical procedure. Surgical delivery (CS) has been associated with increased concentrations of TGF-β2 concentrations [30]. This cytokine plays a key role in wound healing. It is involved in the migration and activation of inflammatory cells in response to surgical trauma, it promotes granular tissue formation, angiogenesis, re-epithelialization and in high concentrations it influences the transition from fibroblast to myofibroblasts, thereby accelerating scar formation. The observed increased levels could reflect this function of TGF-β2 [22,31]. The underlying immunopathology could not be determined in this clinical study and induction of TGF-β by macrophages in response to surgery-induced apoptosis via the activation of the scavenger receptor CD36 cannot be excluded [21].

The indications for surgical delivery were not documented, therefore it is plausible that factors that prompted patients to deliver surgically and often (56%) prematurely, could also have affected the concentrations of the TGF-β2 cytokine. Further systematic research studies of these factors should be conducted in order to better understand why the TGF-β2 protein concentrations are higher in preterm than term colostrum and whether surgical delivery has a confounding effect.

The clinical implications of the observations that TGF-β2 isoform concentration is comparable in term as in preterm colostrum suggest that supplementation of this isoform alone may not mitigate the deleterious consequences of defects in the non-specific immune defenses. However, the association of high concentrations with both preterm colostrum and delivery via caesarean section, that overlapped in our patient cohort mandates careful consideration of the utility of providing supplementary feeding that is fortified with TGF-β2. Until adequate evidence is accrued, it may be prudent to implement early and exclusive breast-feeding by mothers after caesarean section and premature births with colostrum, which may prevent a negative impact of pathogens that often colonize the gastrointestinal tract and may reduce the risk of chronic diseases in this group of patients. There is evidence that the rich nutrients in colostrum and mature milk could mitigate food intolerance, reduce the chances of developing necrotizing enterocolitis, and mitigate growth retardation in the affected preterm infants.

## 5. Summary

We presented TGF-β2 concentrations in human colostrum in every age group of premature babies. We also analyzed TGF-β2 concentration in the breast milk of women after caesarean section and vaginal delivery. Based on the results obtained, we believe that exclusive breastfeeding by mothers after caesarean section and premature births with colostrum containing high TGF-β2 levels may prevent the negative impact of pathogens which often colonize the gastrointestinal tract and it may reduce the risk of necrotizing enterocolitis in premature neonates as well as chronic diseases in the future. These results are an introduction to further research, including the analysis of the composition of the intestinal microflora in this group of patients.

## Figures and Tables

**Table 1:** Inclusion and exclusion criteria.

Inclusion Criteria	Exclusion Criteria
Informed consent to participate in the study	Lack of informed consent to participate in the study
Vaginal delivery	Assisted vaginal delivery
Surgical delivery	Active mother’s infection
Adequate colostrum lactation for the collection of samples	Breast infection/inflammations
Single pregnancy	Chronic antibiotic therapy
	Autoimmune diseases
	Gestational diabetes
	HELLP * syndrome
	Preeclampsia and eclampsia
	Multiple pregnancy
	Smoking

* HELLP (Hemolysis, Elevated Liver enzymes, Low Platelet count).

**Table 2:** Transforming growth factor β (TGF- β2) concentration in human colostrum (HC) after preterm delivery according to weeks of gestation (WG).

	Number of Patients	Mean Gestational Age ± Standard Deviation (SD)	Mean TGF-*β2* Concentration in HC [ng/mL]	SD
Extremely preterm, ≤28 WG	3	27.00 ± 1.73	3685	±1032
Very preterm, 29–32 WG	6	26.50 ± 1.70	4235	±664
Late preterm, 33–36^6/7^ WG	39	34.95 ± 2.94	6026	±622
